# Scalability study on [^133^La]LaCl_3_ production with a focus on potential clinical applications

**DOI:** 10.1186/s41181-024-00292-w

**Published:** 2024-08-15

**Authors:** Santiago Andrés Brühlmann, Martin Walther, Magdalena Kerstin Blei, Constantin Mamat, Klaus Kopka, Robert Freudenberg, Martin Kreller

**Affiliations:** 1 Institute of Radiopharmaceutical Cancer Research, Helmholtz-Zentrum Dresden-Rossendorf, Bautzner Landstraße 400, 01328 Dresden, Germany; 2 Faculty of Chemistry and Food Chemistry, School of Science, Technische Universität Dresden, 01062 Dresden, Germany; 3 National Center for Tumor Diseases (NCT) Dresden, University Hospital Carl Gustav Carus, Fetscherstraße 74, 01307 Dresden, Germany; 4 German Cancer Consortium (DKTK), Partner Site Dresden, Fetscherstraße 74, 01307 Dresden, Germany; 5 Department of Nuclear Medicine, University Hospital Carl Gustav Carus, Fetscherstraße 74, 01307 Dresden, Germany

**Keywords:** TAT, ^133^La, ^225^Ac, Macropa, Targetry, Dosimetry

## Abstract

**Background:**

In recent years, targeted alpha therapy has gained importance in the clinics, and in particular, the alpha-emitter ^225^Ac plays a fundamental role in this clinical development. Nevertheless, depending on the chelating system no real diagnostic alternative has been established which shares similar chemical properties with this alpha-emitting radionuclide. In fact, the race to launch a diagnostic radionuclide to form a matched pair with ^225^Ac is still open, and ^133^La features attractive radiation properties to claim this place. However, in order to enable its translation into clinical use, upscaling of the production of this PET radionuclide is needed.

**Results:**

A study on optimal irradiation parameters, separation conditions and an exhaustive product characterization was carried out. In this framework, a proton irradiation of 2 h, 60 µA and 18.7 MeV produced ^133^La activities of up to 10.7 GBq at end of bombardment. In addition, the performance of four different chromatographic resins were tested and two optimized purification methods presented, taking approximately 20 min with a ^133^La recovery efficiencies of over 98%, decay corrected. High radionuclide purity and apparent molar activity was proved, of over 99.5% and 120 GBq/µmol, respectively, at end of purification. Furthermore, quantitative complexation of PSMA-617 and mcp-M-PSMA were obtained with molar activities up to 80 GBq/µmol. In addition, both ^133^La-radioconjugates offered high stability in serum, of over (98.5 ± 0.3)% and (99.20 ± 0.08)%, respectively, for up to 24 h. A first dosimetry estimation was also performed and it was calculated that an ^133^La application for imaging with between 350 and 750 MBq would only have an effective dose of 2.1–4.4 mSv, which is comparable to that of ^18^F and ^68^Ga based radiopharmaceuticals.

**Conclusions:**

In this article we present an overarching study on ^133^La production, from the radiation parameters optimization to a clinical dose estimation. Lanthanum-133 activities in the GBq range could be produced, formulated as [^133^La]LaCl_3_ with high quality regarding radiolabeling and radionuclide purity. We believe that increasing the ^133^La availability will further promote the development of radiopharmaceuticals based on macropa or other chelators suitable for ^225^Ac.

**Graphical abstract:**

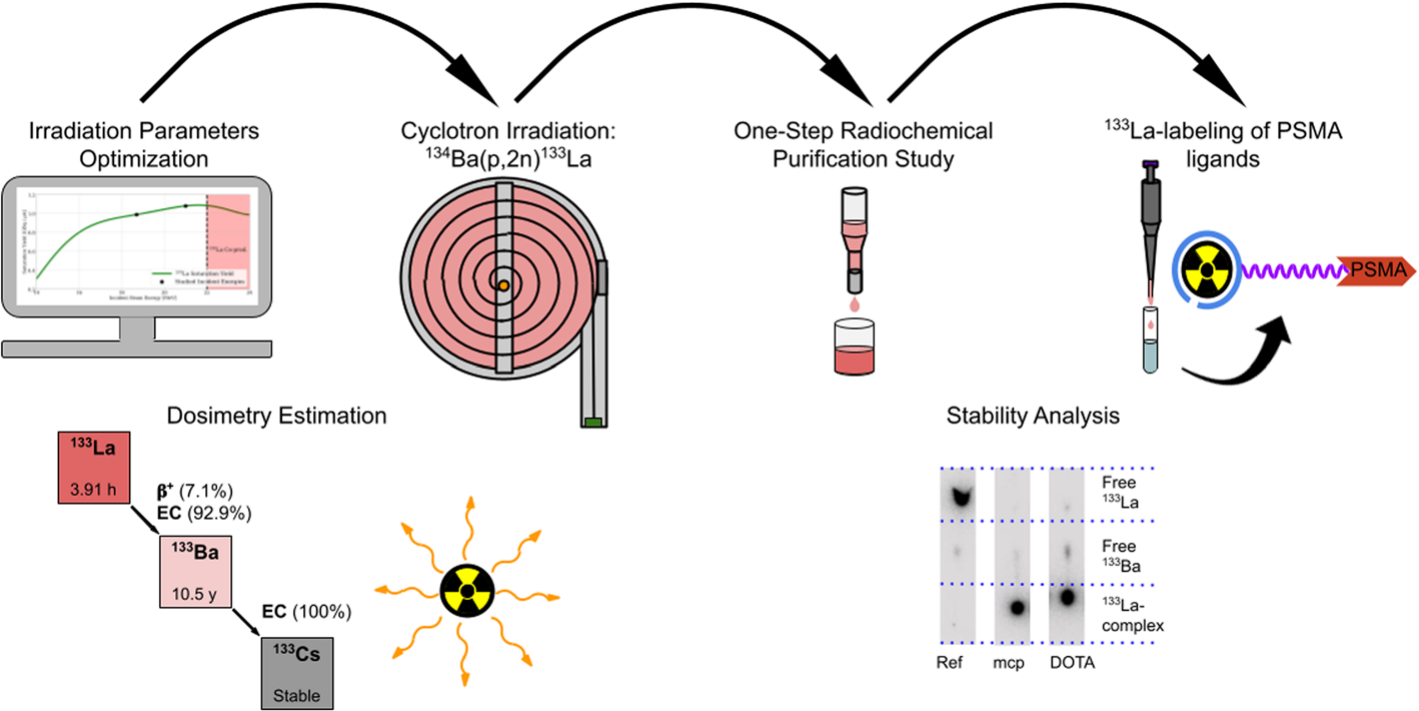

## Introduction

Over the last couple of years, the number of studies regarding targeted alpha therapy (TAT) has increased exponentially (Pallares and Abergel 2022). Radiation comprising a high linear energy transfer (LET), such as α particles and Meitner–Auger electrons (MAE), can inflict a higher damage in targeted cells while sparing the neighboring healthy tissue compared to therapeutically applied β particles (Eychenne et al. 2021; Ku et al. 2019). In fact, both α particles as well as MAE have a shorter particle range as compared with β particles, meaning that less side-effects could be expected when the radionuclide is bound to the right targeting vector, either covalently or in the form of a stable and kinetically inert metal complex. Moreover, α particles and MAE fortunately can efficiently treat micrometastases, normally not detectable by means of noninvasive molecular imaging. In particular, the range of α particles lies between 50 and 100 µm which is enough to induce cell death even when the radiopharmaceutical is not internalized in the cell (Eychenne et al. 2021; Miederer et al. 2024).

There are only a handful of α-emitting radionuclides with potential use in nuclear medicine; from these α-emitters, ^223^Ra took the lead in 2013 when radiopharmaceutical, Xofigo® ([^223^Ra]RaCl_2_) was FDA- and EMA-approved (Gott et al. 2016; Poeppel et al. 2018). However, in later years the attention has turned also to other α-emitters due to their easier coordination chemistry or their potential to covalently bind to the conjugates of interest. Noteworthy is the first actinide ^225^Ac; it became the radionuclide of highest interest for TAT due to its availability mainly from thorium-229 sources, easy chelation chemistry and attractive physical properties (ca. 10 days half-life, decay via emission of four α and two β^−^ particles to ^209^Bi) (Eychenne et al. 2021; Guerra Liberal et al. 2020; Kratochwil et al. 2016). Furthermore, in recent years the ^212^Pb/^212^Bi in vivo generator, with half-lives of 10.6 h and 60.6 min, respectively, has also gained relevance, featuring the emission of an α and two β^−^ particles, although some concern may arise from the “hard” gamma-lines of its progeny thallium-208 (Kokov et al. 2022; Bartoś et al. 2013). In addition, ^149^Tb has also piqued some interest in the radiopharmaceutical community due to its featured single α emission (4.12 h; 16.7% α, 7.1% β^+^ and EC) (Müller et al. 2017; Favaretto et al. 2024). Last but not least, the heavy halogen ^211^At has also generated tremendous interest (7.21 h half-life, single α emission) (Zalutsky and Pruszynski 2011; Choi et al. 2018; Watabe et al. 2022). Unfortunately, despite the huge knowledge acquired with radioiodine, its chemical properties cannot be easily transferred to radioastatine because of the rather weak astatine-carbon bond and the associated different chemical behavior.

The use of appropriate matched pairs of radionuclides is also desired, and while ^212^Pb could be matched with ^203^Pb (McNeil et al. 2021; Banerjee et al. 2019), ^211^At with ^123/124^I (Vaidyanathan et al. 2007), and ^149/161^Tb with ^152/155^Tb (Müller et al. 2012); the race for a suitable diagnostic pair with ^225^Ac is still open. Although ^68^Ga is currently the gold standard of metallic radionuclides for PET imaging in the clinics, due to its relevant differences with ^225^Ac it is far from forming an appealing matched pair. Therefore, pursuit of a more suitable diagnostic counterpart for this α-emitting radionuclide is of high interest. So far, the only true matched pair proposed has been ^226^Ac (29.4 h half-life, 17% EC and 83% β^−^, complex decay scheme including emission of four α particles to long-lived ^210^Pb [half-life 22.2 y]), which has a rather suitable half-life to perform dosimetric calculations, however, since it is a γ-emitter it is only suitable for SPECT imaging (Koniar et al. 2024). Furthermore, the consecutive α decays of its progenies are a matter of dosimetric concern (Nelson et al. 2023). Alternatively, lanthanum radioisotopes have been studied since this element is an excellent surrogate for actinium, with a comparable ionic radii and similar coordination chemistry (Kovács 2020). For that purpose, β^+^-emitters ^132^La, ^133^La and ^134^La have been proposed; the latter being relevant in an in vivo ^134^Ce/^134^La generator (Aluicio-Sarduy et al. 2019; Nelson et al. 2021; Bailey et al. 2020; Bobba et al. 2024). Decay properties of the previous mentioned radionuclides are presented in Table 1 (Nucleus. 2024).

**Table 1:** Imaging radionuclides used and proposed to match α-emitter ^225^Ac

Radionuclide	Half-life/h	Positron mean energy/keV (intensity/%)	Gamma energy/keV (intensity > 1/%)
^68^Ga	1.13	830 (88.9)	1077 (3.22)
^132^La	4.80	1290 (42.1)	464.5 (76)
			567.1 (15.7)
			1909 (9.0)
			663.0 (9.0)
			1031 (7.8) i.a
^133^La	3.91	461 (7.2)	278.8 (2.44)
			302.4 (1.61)
			290.1 (1.38)
			12.3 (1.38)
^134^Ce/^134^La	75.8/0.11	No β^+^/1217 (63.6)	No relevant γ/604.7 (5.0)
^226^Ac	29.4	-	158.0 (17.5)
			230.0 (26.9)
			185.6 (4.8)
			253.5 (5.7)

Moreover, the higher PET imaging quality of ^133^La (despite low positron emission intensity) over ^132^La, and even over ^68^Ga, has been demonstrated by an interesting study using Derenzo phantoms performed by Nelson et al. (2021), which can be explained due to the lower energy of the β^+^-particles emitted by the former. The main challenge regarding ^133^La is its decay to long-lived barium-133, however, this factor could be irrelevant when considering the intense high-energy γ co-emission of ^132^La. Dosimetry studies are still needed to better assess these aspects. Furthermore, in principle, a similar PET quality as that of ^132^La images would be expected for ^134^La since they have a similar positron mean energy emission and thus tissue penetration. Nevertheless, the quality could be worse due to the expected lanthanum decomplexation after the ^134^Ce EC transformation and in fact, this decomplexation has already been observed (Bobba et al. 2024). All in all, the advantage of the ^134^Ce/^134^La in vivo generator lies in its longer half-life and the possibility to perform dosimetry calculations.

On the other hand, due to chemical resemblances of the 4f-elements, ^133^La could also serve as a diagnostic counterpart for some emerging radiolanthanides. In fact, the true theranostic matched pair ^132/133^La/^135^La (half-life 19.5 h, EC 100% i.e. MAE) has raised some interest in pursuit of a new generation of radiopharmaceuticals based on MAE emitters (Aluicio-Sarduy et al. 2019; Nelson et al. 2020; Pedersen et al. 2023; Fonslet et al. 2017). Furthermore, other early lanthanides could also benefit from this PET radionuclide. For this matter, β^−^-emitters ^143^Pr or ^149^Pm have raised some interest as alternatives to ^177^Lu (Sadler et al. 2022) and could be easily paired with ^133^La.

In addition, the concept of matched pair radionuclides could be further extended when considering macromolecular targeting vectors containing different chelators, e.g. antibodies radiolabeled with ^225^Ac (macropa) and ^89^Zr (DFO) (Babeker et al. 2024). In this way, other PET radionuclides would come into play, such as ^52^Mn (Chaple and Lapi 2018), ^55^Co (Lin et al. 2024), ^152^Tb (Müller et al. 2012) or even ^43/44^Sc (Chaple and Lapi 2018) or ^61/64^Cu (Brühlmann et al. 2024; Avila-Rodriguez et al. 2007).

La^3+^ is mandatory to be imbedded into a chelator that can be furnished with a molecular targeting vector for targeted applications in radiopharmacy. The trivalent La^3+^ cation has a low charge density, which is best complexed with oxygen donor-containing ligands. Consequently, encapsulation by multidentate, macrocyclic chelators such as the most used standard ligand 2,2′,2″,2‴-(1,4,7,10-tetraazacyclododecane-1,4,7,10-tetrayl)tetraacetic acid (DOTA) is required. Several chelators have been studied for the complexation of ^225^Ac and their benefits were summarized by Ferrier et al. (2019). Amongst the typical acyclic and macrocyclic chelators, i.e., DTPA, TETA, TETPA, DOTPA and DOTMP, DOTA and its bifunctional analogues showed the highest complexation efficiency and best in vivo stability (Robertson et al. 2018; McDevitt et al. 2002; Singh Jaggi et al. 2005). In addition, interesting results have been obtained for the chelation of [^133^La]La^3+^ and [^225^Ac]Ac^3+^ with bispidine derivatives (Kopp et al. 2023). Although DOTA works well for a high number of radiometals, it is not the best choice for La^3+^ and its therapeutic counterpart Ac^3+^. In this case, macropa, also known as H2bp18c6, came into focus (Thiele et al. 2017). It was originally invented for actinide and lanthanide separation and has entered the field of radiopharmaceutical sciences as it forms highly stable complexes not only with ^225^Ac and ^13x^La, but also with ^213^Bi, ^131^Ba and ^223/4^Ra, which are all superior over the respective DOTA complexes (Reissig et al. 2022, 2020; Brühlmann et al. 2022).

**Fig. 1:**
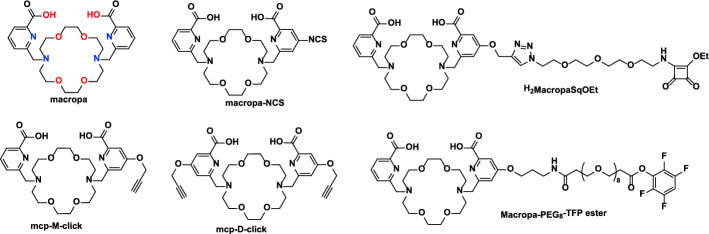
Macropa and examples of functionalized macropa derivatives used for labeling purposes with ^133^La and ^225^Ac

The issue of the [^225^Ac]Ac-DOTA complex lies in its formation kinetics, with slow reaction times and the need for high temperatures, 85–95 °C; the latter limiting its applications for sensitive biomolecules e.g. proteins or antibodies. On the other hand, macropa is able to rapidly form stable complexes with ^225^Ac at room temperature. Such [^225^Ac]Ac-macropa complexes had high in vitro and in vivo stabilities (Thiele et al. 2017; Chappell et al. 2000; Reissig et al. 2021a). Additionally, functionalized and multifunctionalized macropa-chelators for conjugating target molecules were developed in the past years to allow the connection via click chemistry or conventional coupling via NCS or activated esters (Fig. 1) (Reissig et al. 2021a).

Furthermore, conjugates based on the PSMA-617 binding vector and the macropa chelator, namely mcp-M-PSMA and mcp-D-PSMA, were introduced (Reissig et al. 2021a). Those compounds revealed an excellent labeling yield under mild conditions at low concentrations and showed suitable long-term stabilities (Reissig et al. 2022, 2021a). As a proof of concept, mcp-M-PSMA has been also radiolabeled with ^133^La under the same conditions used for labeling with ^225^Ac, obtaining quantitative complexation with [^133^La]La-mcp-M-PSMA molar activities of 50 GBq/µmol (Brühlmann et al. 2022).

Lanthanum-133 has been produced from natural and enriched barium targets, via the ^134^Ba(p,2n)^133^La and the ^135^Ba(p,3n)^133^La nuclear reactions. First results using a natural metallic barium target (200 mg) using 22 MeV proton irradiations at a current of 20 µA for 25 min resulted in activity yields of up to 231 MBq ^133^La and 166 MBq of ^135^La as the main byproduct (Nelson et al. 2020). Later on, the same group further improved the ^133^La/^135^La ratio by using a 200 mg [^135^Ba]BaCO_3_ enriched target irradiated with 23.8 MeV protons at 10 µA for 10 min leading to 214 MBq of ^133^La and 28 MBq of ^135^La (Nelson et al. 2021). However, starting from ^135^Ba, the relatively high ^135^La co-production is unavoidable via the (p,n) nuclear reaction. Furthermore, in our previous study we presented the first results on ^133^La production starting from a 30 mg enriched [^134^Ba]BaCO_3_ target, leading to activities of up to 1.9 GBq of ^133^La with radionuclide purity of 99.5% after 60 min irradiations at 15 µA with 18.7 MeV protons (Brühlmann et al. 2022).

**Fig. 2:**
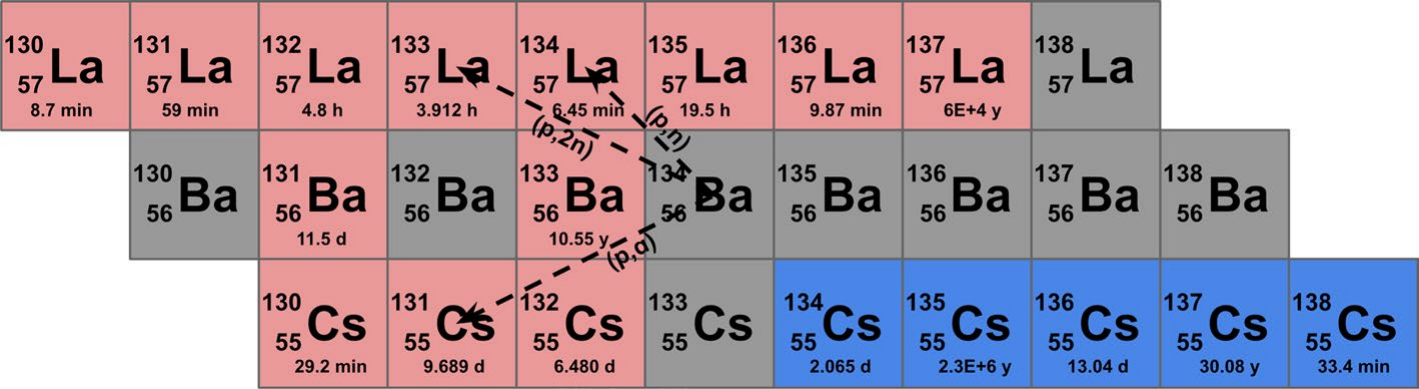
Simplified scheme of nuclear reactions taking place under proton bombardment of a barium target with a cross section of over 1 mb. All three nuclear reactions shown for ^134^Ba also occur for the other stable barium isotopes, however in lesser extent due to their lower enrichment levels

Contrariwise, when bombarding barium targets with proton energies below 22 MeV the (p,n), (p,2n) and (p,α) nuclear reactions take place for each isotope, and their contribution is a function of the level of target material enrichment. In particular, these (p,xn) nuclear reactions have a cross section between 600 and 1100 mb, while the corresponding for the (p,α) nuclear reaction only lies in the 1–10 mb range (TENDL 2019). In Fig. 2 a simplified scheme of these nuclear reactions is shown.

In this work, we aim to further optimize the targetry and target chemistry involved in lanthanum-133 production via the ^134^Ba(p,2n)^133^La nuclear reaction. For that purpose, we started by studying different energy windows and proton currents for the target irradiation as well as the target configuration stability for prolonged irradiations. Moreover, we compare the separation performance of four different commercially available chromatographic resins and optimize the elution parameters. In this context, we perform the quality assessment of the product [^133^La]LaCl_3_ to assess the radioactive and stable impurities. Additionally, we execute an activity demand estimation to show the viability of a safe and reliable lanthanum-133 production route which is transferable into the clinic.

## Methods

### Materials

Solutions used consisted of ultrapure 30% hydrochloric acid (Merck KGaA, Darmstadt, Germany) and ultrapure 69% nitric acid (Roth GmbH, Karlsruhe, Germany) diluted with deionized milli-Q® water. Ammonium acetate 99.999% trace metals basis (Sigma-Aldrich, Schnelldorf, Germany) was used to prepared the buffer solutions. In addition, the following chromatographic resins were acquired: 1 mL pre-packed TK221 cartridge (TrisKem, Bruz, France), 1 mL pre-packed TK222 cartridge (TrisKem), 1 mL pre-packed branched diglycolamide–DGA resin (TrisKem) and normal DGA resin (TrisKem).

Enriched [^134^Ba]BaCO_3_ was purchased from Isoflex (San Francisco, CA, USA), with isotopic composition as presented in Table 2 (supplier specification).

**Table 2:** Isotopic composition of the [^134^Ba]BaCO_3_ enriched target material, as specified by supplier

Isotope	^130^Ba	^132^Ba	^134^Ba	^135^Ba	^136^Ba	^137^Ba	^138^Ba
Content [%]	< 0.01	< 0.01	88.10 ± 0.40	5.36	1.21	1.07	4.26

PSMA-617, macropa and mcp-M-PSMA were prepared according to the literature (Reissig et al. 2021a). Human Serum was purchased from Sigma-Aldrich (St. Louis, MO, USA).

### Dosimetry and injected activity estimation

A rough estimation of the ^133^La activity needed for PET acquisition in humans was determined from ^68^Ga and ^18^F activity amounts usually used for patient injections. Since the purpose of ^133^La is to provide a suitable matched pair for α-emitting ^225^Ac, first approximations were made starting with prostate-specific membrane antigen (PSMA) radioligands, i.e. [^18^F]F-PSMA-1007, [^68^Ga]Ga-PSMA-11 and [^68^Ga]Ga-PSMA-I&T. PET acquisition is performed 1–1.5 h p.i. with an usual scan time of around 20 min. In addition, average injected activities of 200 MBq, 200 MBq and 132 MBq of [^18^F]F-PSMA-1007, [^68^Ga]Ga-PSMA-11 and [^68^Ga]Ga-PSMA-I&T, respectively, have been reported as patient doses (Awenat et al. 2021; Sachpekidis et al. 2016; Strauss et al. 2021; Hennrich and Eder 2021; Cytawa et al. 2019). Moreover, for the sake of the estimation it was assumed, that the ^18^F-, ^68^Ga- and ^133^La-radiolabeled tracers would present similar target accumulation and pharmacokinetics. With all these strong assumptions, the needed integrated positron rate P would be the same for the different radionuclides and can be calculated with Eq. 1, where *A*_*inj*_ stands for the injected activity, *Y*_*p*_ the positron branching ratio, *t*_*dec*_ is the acquisition start time p.i., *T*_1/2_ the radionuclide half-life and *t*_*scan*_ the PET acquisition time. In addition, the first term between brackets represents the activity at the start of the PET scan (i.e. *t*_*dec*_ p.i.), whereas the second term scale this activity to the number of positron emissions occurring during the time of the scan.

1 $$P={\int }_{0}^{{t}_{scan}}A\times {Y}_{p}dt=\left[{ A}_{inj}\times {e}^{-ln\left(2\right)\frac{{t}_{dec}}{{T}_{1/2}}}\right]\times \left[{Y}_{p}\times \frac{{T}_{1/2}}{ln\left(2\right)}\times \left(1-{e}^{-ln\left(2\right)\frac{{t}_{scan}}{{T}_{1/2}}}\right)\right]$$

For the ^133^La-labeled compounds, also an accumulation time of 1 h was used, however, the acquisition time was extended to 1 h in order to reduce the activity injection. In particular, 1 h is the maximal tolerable time that can be considered for a PET acquisition.

In addition, the absorbed dose in different organs and the whole-body effective dose of a homogeneous biodistribution without any specific accumulation and without excretion has been carried out using IDAC-Dose 2.1 (Andersson et al. 2017). In this case, not only the dose provided by ^133^La but also the radioactive daughter ^133^Ba was calculated. For the latter radionuclide, the effective dose after 1 year was also determined.

### Target preparation

**Fig. 3:**
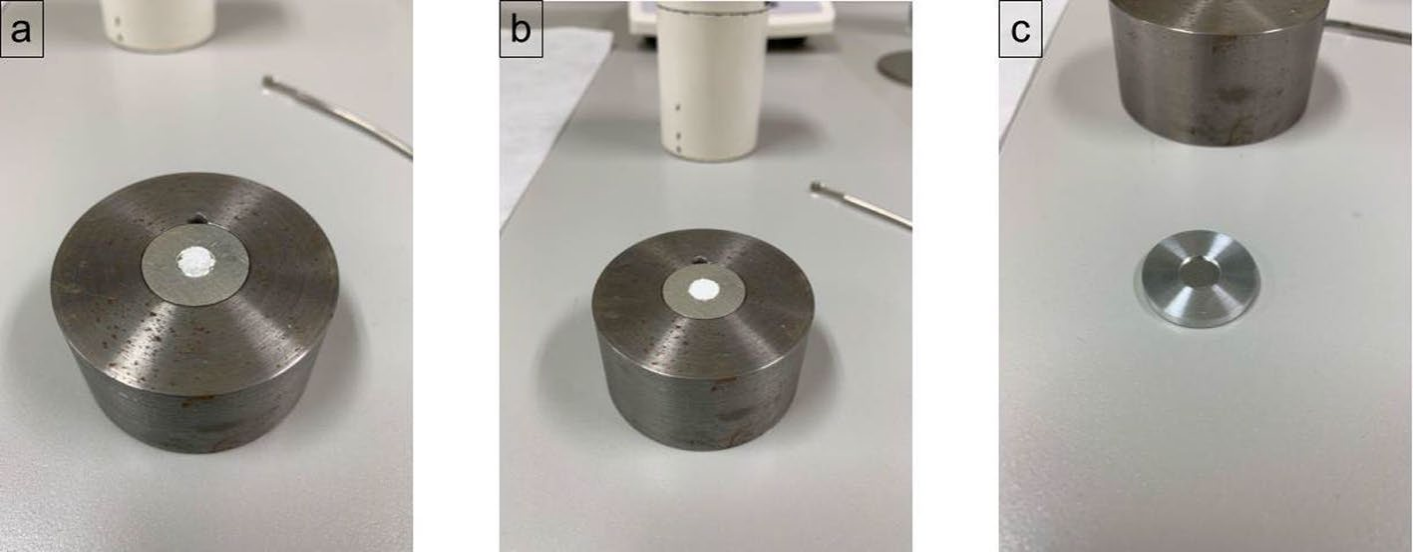
Loading of [^134^Ba]BaCO_3_ powder into a silver backing and an aluminum foil. **a** 25 mg of [^134^Ba]BaCO_3_ powder loaded into the silver backing. **b** [^134^Ba]BaCO_3_ powder arranged before capping the target. **c** Target caped with a 25 μm aluminum foil

Silver and aluminum discs (22 mm diameter, 2 mm thickness) with a centered deepening (9 mm diameter and 0.3 mm depth) were filled with 25 mg of either fresh or recycled [^134^Ba]BaCO_3_ and covered with a 25 µm aluminum foil. The target was pressed with a hydraulic press and caped with an aluminum disc with an opening (10 mm diameter) for the proton beam. In Fig. 3, a typical loading of the [^134^Ba]BaCO_3_ target is presented.

Aluminum was selected as an alternative to silver due to its lower activation, which enables it to be re-used within 2–3 weeks for a new irradiation. Furthermore, thermal simulations proved no significative difference in target temperatures between the two backings, which was later confirmed with successful target irradiation tests.

### Irradiation parameters optimization

The described targets were irradiated using the 90° solid state target configuration of the TR-Flex (ACSI—Advanced Cyclotron System Inc) cyclotron installed at the HZDR (Helmholtz-Zentrum Dresden-Rossendorf). Target cooling is performed with water on the backside (6 L/min, 20 °C) and with helium at the front (300 L/min, 20–25 °C). The proton beam profile has been previously described with a full width to half maximum, FWHM, of 12–14 mm at an energy range of 14–30 MeV (Kreller et al. 2020).

**Fig. 4:**
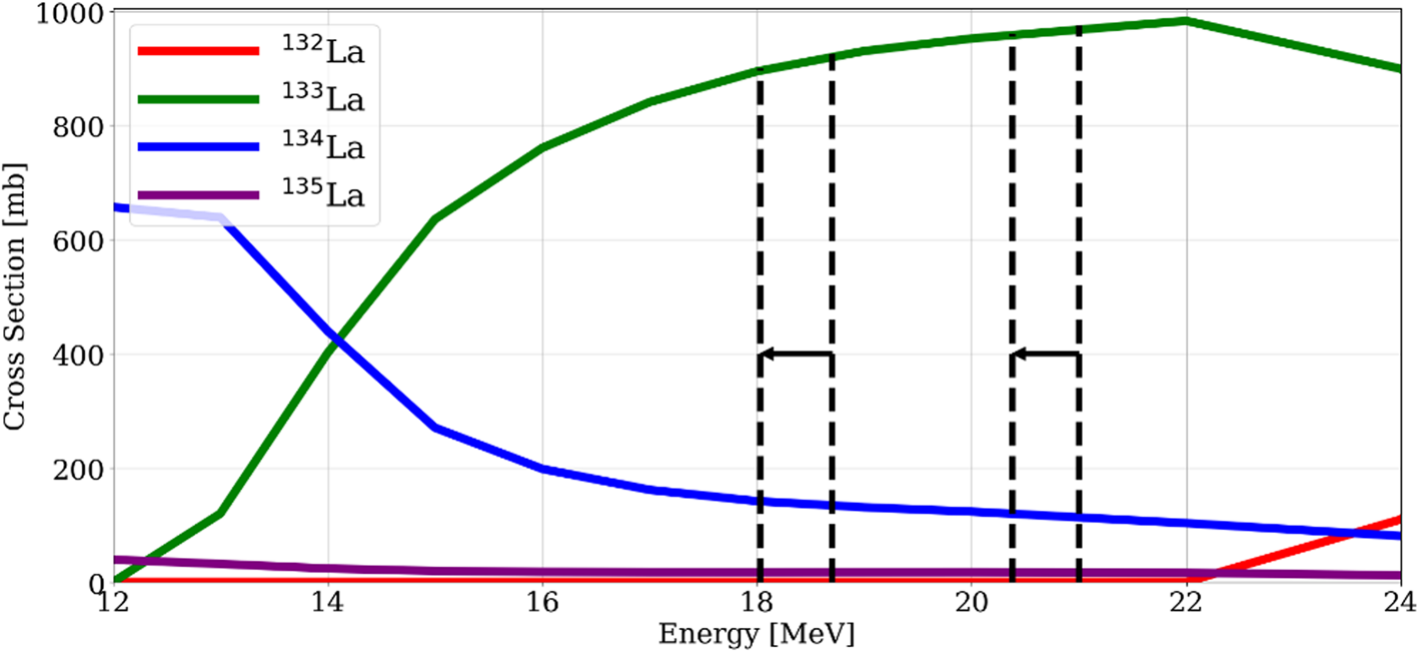
Summary of the cross sections of ^13x^Ba(p,xn)^13x^La nuclear reactions weighted with the isotopic composition of the enriched [^134^Ba]BaCO_3_ target. The energy windows used for the irradiation are located inside the dotted lines

Proton energies of (24.0 ± 0.1) MeV and (22.0 ± 0.1) MeV were extracted from the TR-Flex cyclotron and reduced with a 600 µm aluminum degrader in front of the target. The energy in the target was estimated to be degraded from (21.0 ± 0.1) MeV to (20.4 ± 0.1) MeV and from (18.7 ± 0.1) MeV to (18.0 ± 0.1) MeV, respectively. The cross sections of the nuclear reactions leading to lanthanum radioisotopes from a barium target (TENDL 2019) were weighted for the isotopic composition (Table 2), and the energies degraded in the targets are presented in Fig. 4.

Irradiations at higher energy (E_in_ = 21 MeV) were performed using the silver backing to fully stop the protons within the solid target, while both aluminum and silver backings were used for the lower energy window (E_in_ = 18.7 MeV).

Proton currents ranged from 15 to 60 µA. Thermal simulations using the COMSOL platform (COMSOL 2021) motivated the increase in the proton currents to maximize radionuclide production. First irradiations started with 15 µA and the current was ramped up to 60 µA, in 5 µA steps.

Irradiation times ranged from 15 to 45 min, depending on radionuclide demand. A 2-h, 60 µA and 18.7 MeV irradiation was performed beforehand to test the target configuration stability.

### Radiochemical purification

Radiochemical separation of the ^133^La from the bulk barium carbonate followed a one-step chromatographic purification, similar to a previously published method (Nelson et al. 2020; Brühlmann et al. 2022). A 2 mL diglycolamide-based resin afforded satisfactory separation, however, the elution volume could still be optimized. For this purpose, four different resins were tested: 1 mL branched and normal DGA in addition to TrisKem TK221 (B-grade) and TK222 resins.

After irradiation, the 25 mg of [^134^Ba]BaCO_3_ was dissolved in 2 mL of 1 M HNO_3_ and loaded onto the previously mentioned cartridges. The cartridges were then washed with 6 × 3 mL of 3 M HNO_3_ to diminish [^133^La]La^3+^ breakthrough, followed by a deacidification step where the cartridge was washed with 2.5 mL of 0.5 M HNO_3_. Elution of the ^133^La was performed with 12 × 0.5 mL of 0.05 M HCl.

After characterization of the four chromatographic resins, the two more promising candidates were selected and the acid concentration and volumes adapted to optimize the radiochemical separation. The defined parameters are presented in the results.

Additionally, the barium-containing fractions are collected and left to decay. After ca. 2 months, the [^134^Ba]BaCO_3_ is recovered by precipitation of the barium carbonate. For that purpose, 10–15 recovered [^134^Ba]Ba(NO_3_)_2_ fractions in nitric acid were collected and neutralized by addition of 3 M sodium hydroxide, followed by addition of 1 M ammonium carbonate solution until no further precipitation of the barium carbonate occurs. The white precipitate is then washed 5 times with 10 mL of water obtaining a wash solution free of nitrate ions. The [^134^Ba]BaCO_3_ is dried at 120 °C until constant weight.

### Product characterization

#### Radionuclide purity

Radionuclide characterization has been carried out by high-resolution gamma spectroscopy using an energy- and efficiency-calibrated Mirion Technologies (Canberra, Australia) CryoPulse 5 HPGe detector. Activities are automatically calculated with the Genie2000 software (V. 3.4.1). Fractions containing between 100 and 800 kBq were measured for 10 min shortly after (within 1 h) the end of purification (EOP). In particular, 200 µL of the barium-containing fractions were filled into a tube with calibrated geometry for the gamma spectroscopy measurement, while 50–100 µL of the deacidification fraction or 1–5 µL of the [^133^La]LaCl_3_ eluent were filled in the same tubes and filled with water to reach the 200 µL. Moreover, a last probe containing between 100 and 250 MBq ^133^La, i.e. 100–200 µL of the product solution, was measured for 1 h after 48–72 h to perform radionuclide purity (RNP) analysis. Dead-time was always ensured below 5%.

#### Molar activity

Molar activity of the produced [^133^La]LaCl_3_ was determined by ICP-MS measurements in combination with activity quantification. From the product solution, 200 µL containing 150–250 MBq ^133^La were saved for the analysis. Barium-134 ([^134^Ba]Ba^2+^), lanthanum (La^3+^), aluminum (Al^3+^), iron (Fe^3+^), copper (Cu^2+^), zinc (Zn^2+^) and lead (Pb^2+^) content was looked for. ICP-MS measurements were performed by VKTA e.V. (Radiation Protection, Analytics and Disposal-Dresden, Germany) with a ThermoFischer Element 2.

Apparent molar activity (AMA) was quantified by titration with the macropa chelator in combination with radio-TLC. 3–10 µL [^133^La]LaCl_3_ (4–7 MBq, approximately 10 MBq at EOB) were added to a buffered solution (ammonium acetate 200 mM, pH 6, total volume 100 µL) containing different chelator concentrations: 0.20 µM, 0.40 µM, 0.80 µM and 1.60 µM. These solutions were mixed at 500 rpm for 15 min at room temperature. The formed [^133^La]La-macropa complex was analyzed using silica gel coated aluminum plates and developed with 50 mM EDTA as eluent. While the free lanthanum runs to the front, the complex remains at the start.

### Radiolabeling of PSMA ligands and stability study

**Fig. 5:**
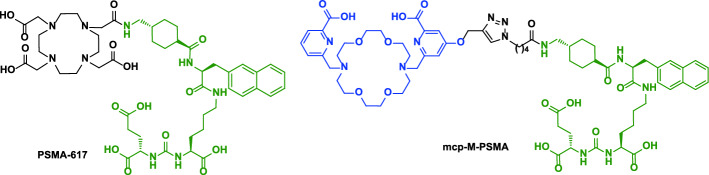
Chemical structure of PSMA-617 and mcp-M-PSMA

Radiolabeling of PSMA ligands was performed 2 h after EOP and was carried out similarly to the test radiolabeling with macropa, but using higher ^133^La activities. From the product solution, 200 MBq of [^133^La]LaCl_3_ (100–150 µL) were mixed with 2.5 nmol and 5 nmol of either PSMA-617 or mcp-M-PSMA (Reissig et al. 2021a) in a buffered solution (2.5 µL or 5 µL of a 1 mM solution of PSMA-conjugates, ammonium acetate 200 mM pH 6, total volume 300 µL). The obtained solution was mixed at 500 rpm for 30 min at 90 °C. Radiochemical yield of both complexes was assessed by radio-TLC following the same method as for the test radiolabeling. Chemical structure of both PSMA conjugates are presented in Fig. 5.

To assess the serum stability of both radiolabeled compounds, 75 µL of the ^133^La-PSMA radioconjugates, accounting for ca. 50 MBq ^133^La, were added to 75 µL of PBS buffer pH 7 and 150 µL of human serum and mixed at 500 rpm for 24 h at 37 °C. Silica gel plates were spotted after 1 h, 4 h and 24 h to evaluate the evolution of the complex stability. Each ^133^La-PSMA radioligand and its molar activity was tested thrice.

## Results

### Dosimetry and injected activity estimation

Taking the administered activities of similar radiopharmaceuticals as a starting point, it was estimated that an activity between 350 and 750 MBq of ^133^La-PSMA ligand would be needed to perform PET imaging. It should be mentioned that the time needed for target accumulation may be different and thus the activities modified. Furthermore, imaging at later time points, i.e. for dosimetry calculations, could be performed after administration of higher ^133^La activities. Nevertheless, the effective dose resulting from these activities should be more accurately estimated.

Furthermore, the full body effective dose per activity unit of ^133^La and ^133^Ba are shown in Table 3. In particular, the effective dose after 1 year and the total effective dose are included.

**Table 3:** Effective dose of a homogenous ^133^La-biodistribution without excretion

	^133^La	^133^Ba (1 year)	^133^Ba	^133^La + ^133^Ba (1 year)
Effective dose/mSv/MBq	5.25 × 10^−3^	6.54 × 10^−4^	1.01 × 10^−2^	5.90 × 10^−3^
Effective dose/mSv from 350 MBq	1.84	0.23	3.54	2.06
Effective dose/mSv from 750 MBq	3.94	0.48	7.58	4.42

Considering the necessary ^133^La estimated activities for imaging and the effective dose per activity unit, a first rough whole-body effective dose ranging from 5.4 to 11.3 mSv can be calculated. Actually, after 1 year the effective dose accounts for roughly 2.1–4.4 mSv.

### Irradiation parameters optimization

**Fig. 6:**
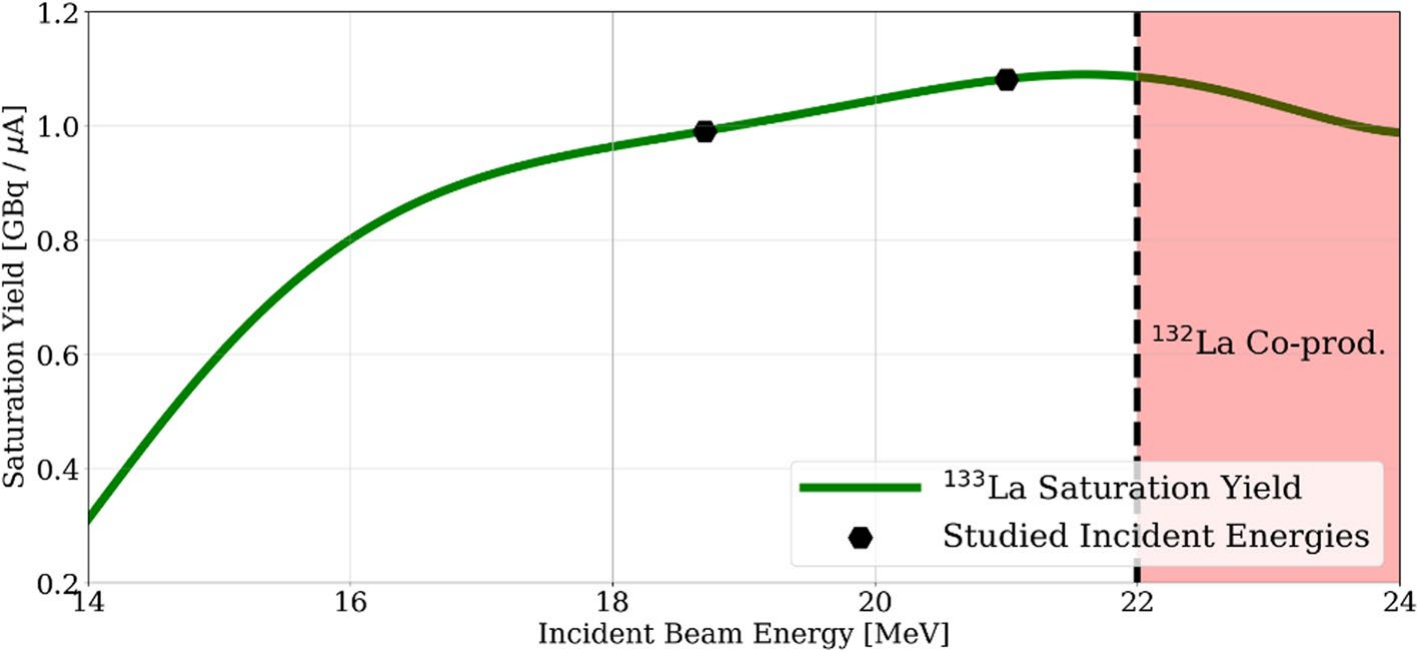
Theoretical saturation yield of the studied 25 mg [^134^Ba]BaCO_3_ target as a function of the incident proton energy. In red the incident energies provoking ^132^La co-production. The hexagons show the studied incident energies of the irradiations

Lanthanum-133 theoretical saturation yields were calculated as previously reported (Brühlmann et al. 2022), considering the [^134^Ba]BaCO_3_ target thickness and enrichment. In Fig. 6, the ^133^La saturation yield as a function of the incident proton energy is presented. It is noteworthy that this yield maps almost perfectly the cross section of the reaction, presented in Fig. 4, due to the small thickness of the studied target. However, since the stopping power of protons at higher energies is lower, some minor distortion is also observed.

The investigated 25 mg of [^134^Ba]BaCO_3_ target presented a saturation yield of (680 ± 50) MBq/µA (n = 3) for the estimated (21.0 ± 0.1) MeV to (20.4 ± 0.1) MeV window, while (580 ± 90) MBq/µA (n = 12) for the estimated (18.7 ± 0.1) MeV to (18.0 ± 0.1) MeV window. These values contrast with the theoretical saturation yields of 1080 MBq/µA and 990 MBq/µA, respectively, calculated from the simulation.

The 15 min proton irradiations at a 60 µA current and 18.7 MeV led to an ^133^La activity of 1.6 GBq at EOB. In contrast, the 2-h irradiation with the same proton energy and current, produced 10.7 GBq of ^133^La at EOB corresponding to a saturation yield of 600 MBq/µA, which is in agreement within the uncertainty range of the previous presented yields. This long irradiation was performed once to confirm the target configuration stability and thus validate ^133^La production upscaling.

### Radiochemical separation

**Fig. 7:**
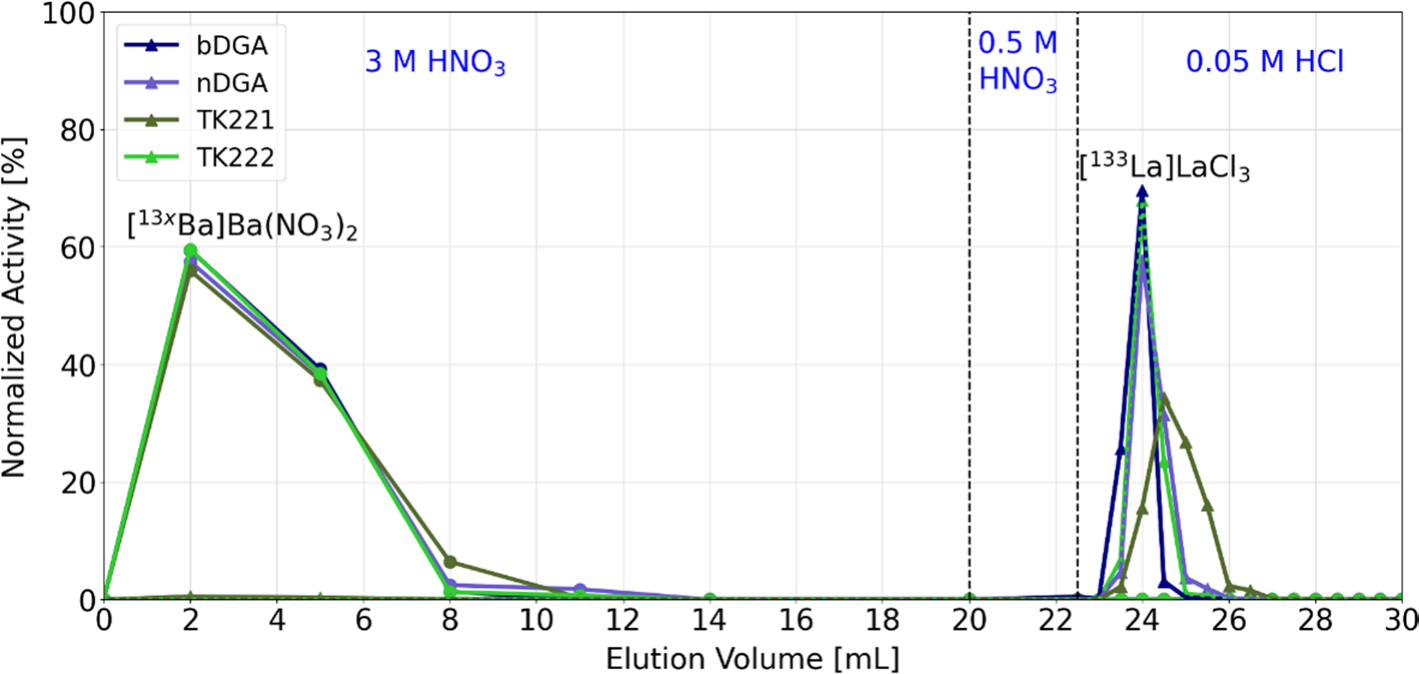
Elution profile of the barium-lanthanum radiochemical separation. The performance of four different chromatographic resins was studied: normal and branched DGA and TK221-B and TK222 resins

Before target workup, the disc is left to decay at least 40 min to reduce the ^134^La content. The target is then opened and the irradiated [^134^Ba]BaCO_3_ is dissolved in 2 mL of 1 M HNO_3_ and loaded onto the 1 mL chromatographic resin cartridge for the radiochemical purification. The four chromatographic resins studied, bDGA, nDGA, TK221-B and TK222 resulted in a successful separation of the product ^133^La from the bulk [^134^Ba]BaCO_3_ target material. However, some differences in the lanthanum breakthrough during the target loading, the washing and particularly in the de-acidification steps were observed. Furthermore, the ^133^La elution volumes were different for each resin. The elution profile of the four chromatographic resins (each resin used only once, two independent tests) is presented in Fig. 7.

The sharpest ^133^La elution curves were obtained with the bDGA and the TK222 resins. In 1 mL of the 0.05 M HCl product solution over 94% of the ^133^La activity was obtained when using the bDGA and TK222 resins, 84% with the nDGA and only 61% with the TK221-B. While for the bDGA the activity peak was obtained in product fractions 2 and 3, for the TK222 resin the most relevant fractions were the number 3 and 4, each 0.5 mL. Based in this elution characteristics, the bDGA and TK222 resins were further investigated.

**Fig. 8:**
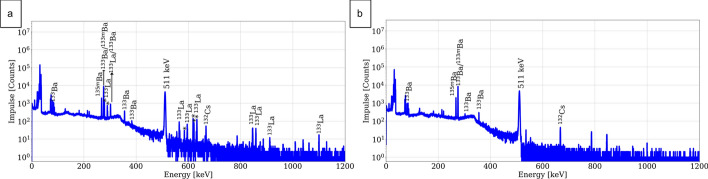
Representative gamma spectra of the barium fractions obtained for the two optimized separation methods. **a** Using the bDGA resin. **b** Using the TK222 resin

Moreover, lanthanum breakthrough was already observed during the target solution loading and washing fractions for both DGA resins and the TK221-B resin, while it was first detected during the de-acidification step for the TK222 resin. Nevertheless, the total breakthrough was in any case not higher than 1% of the total ^133^La activity. To illustrate the lanthanum breakthrough, in Fig. 8 gamma spectroscopy analyses of the barium fraction using both the bDGA and TK222 resins are shown.

**Fig. 9:**
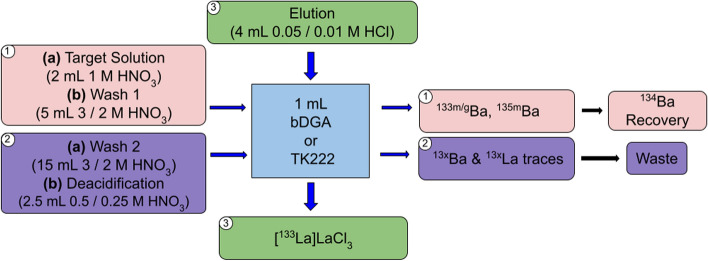
Scheme of the two optimized ^133^La radiochemical purification methods from the [^134^Ba]BaCO_3_ targets, based on branched DGA and TK222 resins

Since no ^133^La breakthrough had been detected for the TK222 resin, it was decided to reduce the nitric acid concentration of the washing and de-acidification fractions. In addition, the ^133^La elution was performed with 10 mM HCl. The adapted acid concentrations as well as the elution volumes used for both purification methods are presented in Fig. 9.

The whole radiochemical separation takes 20 min, and with both methods yielding over 98% of the loaded ^133^La activity obtained in 4 mL of diluted hydrochloric acid (decay corrected). The elution profile of the TK222 resin showed no major difference to that presented in Fig. 7 when reducing the concentration of the acids used.

### Product characterization

#### Radionuclide purity

**Fig. 10:**
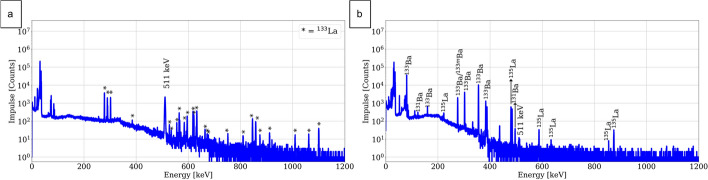
Representative gamma spectra of the product [^133^La]LaCl_3_ fraction. **a** Shortly after EOP **b** 72 h after EOP

Radionuclide characterization of the [^133^La]LaCl_3_ fractions obtained from the four different resins was performed shortly after EOP and after at least 2 days decay. In these product fractions, no major qualitative difference was observed. For illustration representative gamma spectra of the TK222 product solution, shortly after EOP and after 3-day decay, are shown in Fig. 10.

RNP of all the product fractions proved to be higher than 99.6% at EOB. Since longer decay times before target work up were needed on some days due to routine work of the cyclotron, the RNP at EOP cannot be directly compared. The radionuclide impurities detected in the product fractions obtained with the different resins are quantified in Table 4.

**Table 4:** Radionuclide impurities detected in the product [^133^La]LaCl_3_ solution obtained from the different chromatographic resins

Chrom. Resin	^135^La (%)	^135m^Ba (%)	^133m^Ba (%)	^133^Ba (%)	^131^Ba (%)
bDGA	< 0.38	< 3.7 × 10^−4^	< 1.6 × 10^−3^	< 1.2 × 10^−5^	< 2.5 × 10^−5^
nDGA	< 0.38	< 2.6 × 10^−4^	< 1.5 × 10^−3^	< 2.5 × 10^−5^	< 5.0 × 10^−5^
TK221	< 0.38	< 3.1 × 10^−4^	< 1.4 × 10^−3^	< 3.3 × 10^−5^	< 5.8 × 10^−5^
TK222	< 0.38	< 3.3 × 10^−4^	< 1.3 × 10^−3^	< 1.4 × 10^−5^	< 3.3 × 10^−5^

The main radionuclide impurity is ^135^La and thus is not dependent on the radiochemical purification method. Moreover, other radiobarium isotopes in minor extent were also quantified. In particular, ^133^Ba content is calculated from the ^133m^Ba activity detected and cannot be directly quantified due to the huge amount of ^133^La which decays straight to the ground state of the former radionuclide.

It was observed that ^133^La decays to the ^133^Ba ground state resulting in an activity factor inversely proportional to their half-lives, as expected. After ten ^133^La half-lives (ca. 40 h), for every 23.6 MBq of ^133^La, ca. 1 kBq of ^133^Ba has been quantified, which is consistent with the theoretical ratio calculated from the half-lives of both radionuclides.

#### Molar activity

Molar activity and content of other metallic ions in solution was assessed in terms of ICP-MS measurements. For that purpose, [^133^La]LaCl_3_ fractions of four different ^133^La production batches, two purified with the bDGA cartridge and two using the TK222 cartridge, were measured. The ^133^La activity was assessed by gamma spectroscopy and the activity concentration was therefore calculated. Results of the ICP-MS measurements are included in Table 5.

**Table 5:** Stable metallic content of the [^133^La]LaCl_3_ product fraction determined by ICP-MS means for both optimized radiochemical purification methods. The activity concentration of the fractions is also included

Resin	Activity Conc. (@EOP)/GBq/mL	La^3+^/ppb	[^134^Ba]Ba^2+/^ppb	Pb^2+^/ppb	Cu^2+^/ppb	Zn^2+^/ppb	Fe^3+^/ppb	Al^3+^/ppb
bDGA	0.79	144	3520	1360	10	330	12	40
	1.16	105	1420	31,500	13	240	12	54
TK222	1.22	183	760	215	10	290	12	42
	0.94	43	1060	1480	8	140	15	99

The determined molar activities are consistent between the two methods and lie between 760 and 3000 GBq/µmol at EOP. The stable lanthanum content can be attributed to the starting target material, where ca. 1.2 ppm of La where quantified. Another source of lanthanum is the nuclear reaction ^138^Ba(p,n)^138^La also occurring during the proton irradiation.

Furthermore, a barium separation factor in the range of 6.2 × 10^3^ to 1.5 × 10^4^ for the bDGA resin was quantified, while for the TK222 resin this value was between 2.1 × 10^4^ and 2.9 × 10^4^. It should be remarked that ^134^Ba accounts only for 88% of the total barium, which is this isotope enrichment level.

Aluminum concentration in product [^133^La]LaCl_3_ is quite low considering the aluminum backing and foil used. On the other hand, the content of lead results relatively high and comparable to that of barium. Both barium and lead can directly interfere in the ^133^La radiolabeling. In addition, a comparable complex formation of macropa with Cu^2^^+^, Zn^2^^+^, Fe^3^^+^, and Al^3^^+^ is not known.

Complexation of over 95% was obtained for macropa concentrations of 0.80 µM regardless of the purification method, while substantial differences were observed at 0.40 µM. The highest AMA were quantified for the product obtained using the TK222 resin, with values of more than 240 GBq/µmol, while for the product of the bDGA cartridge over 120 GBq/µmol was determined, EOB corrected.

### Radiolabeling of PSMA ligands and stability study

**Fig. 11:**
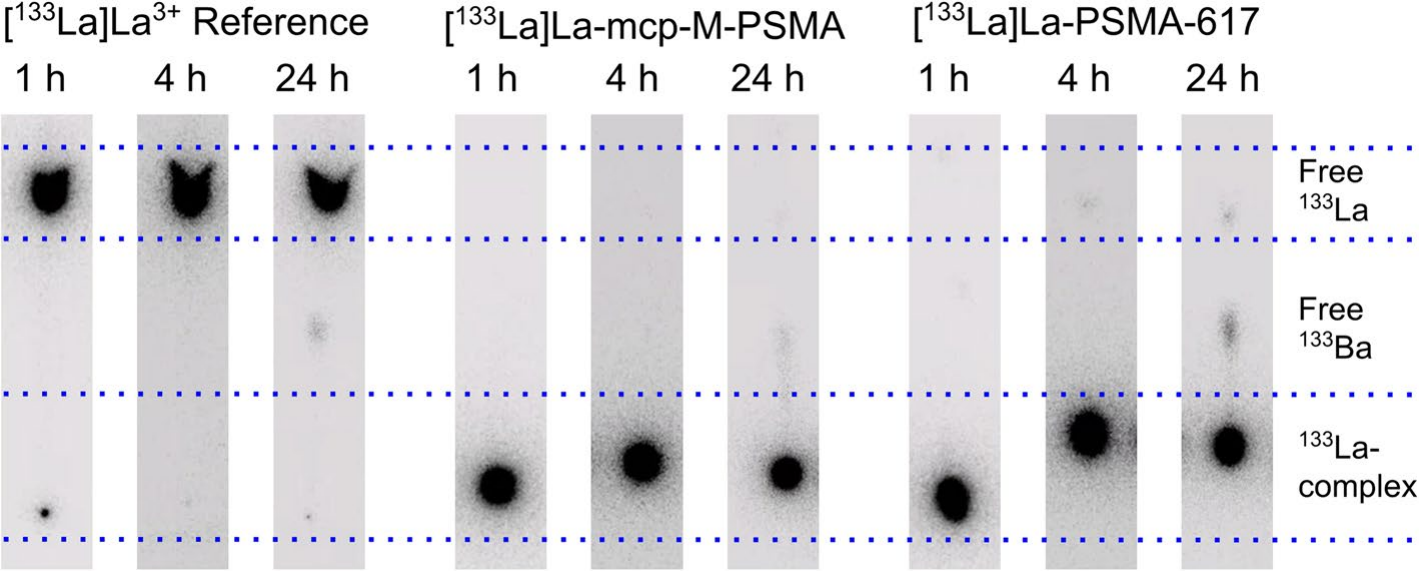
Human serum stability results for [^133^La]La-PSMA-617 and [^133^La]La-mcp-M-PSMA (80 GBq/µmol) after 1 h, 4 h and 24 h. Uncoordinated [^133^La]LaCl_3_ is included as reference

Lanthanum-133 activities in the range of clinical applications were successfully used for labeling of PSMA-617 and mcp-M-PSMA at molar activities of 40 GBq/µmol and 80 GBq/µmol. Nearly quantitative complexation, with radiochemical yields (RCY) of over 99.5% were obtained for both radioconjugates (RCY presented in Table 6). The human serum stabilities of both [^133^La]La-PSMA-617 and [^133^La]La-mcp-M-PSMA were determined after 1 h, 4 h and 24 h. Representative radio-TLC analyses at the different timepoints are shown in Fig. 11, while the evaluated results are presented in Table 6.

**Table 6:** Radiochemical yield and stability in human serum of [^133^La]La-PSMA-617 and [^133^La]La-mcp-M-PSMA ligands

Radioligand	Molar activity/GBq/µmol	Radiochemical yield/%	HS stability 1 h/%	HS stability 4 h/%	HS stability 24 h/%
[^133^La]La-PSMA-617	40	99.7	99.37 ± 0.12	99.40 ± 0.01	97.37 ± 0.17
	80	99.6	99.57 ± 0.12	99.60 ± 0.08	98.5 ± 0.3
[^133^La]La-mcp-M-PSMA	40	100	100 ± 0	100 ± 0	99.57 ± 0.25
	80	99.7	99.80 ± 0.01	99.83 ± 0.08	99.20 ± 0.08

Furthermore, the presence of uncoordinated (free) [^133^Ba]Ba^2+^ can be observed in Fig. 11, and has been quantified. After 24 h in human serum, the amount of free radiobarium amounted for 1.1% of the total radioactivity in the uncoordinated reference, while for the [^133^La]La-PSMA-617 and [^133^La]La-mcp-M-PSMA radioligand batches (1.1 ± 0.3)% and (0.55 ± 0.04)%, respectively.

## Discussion

### Dosimetry and injected activity estimation

From previous publications, injected ^68^Ga activities of PSMA-radioligands in humans are usually a factor of 7.5 higher than that used for animal imaging (Awenat et al. 2021). In addition, first animal experiments using 33–50 MBq of [^133^La]La-PSMA-I&T have been reported (Nelson et al. 2021). Taking this into consideration, the estimated ^133^La activity range calculated of 350–750 MBq is in accordance with these reported factors.

The ^133^Ba activity resulting from 750 MBq of ^133^La may arise intrigue, which accounts for roughly 30 kBq, nevertheless, although in our model no excretion was considered, in reality over 80% of the injected activity would be excreted within 24 h (Reissig et al. 2021b). In this case, the remaining ^133^Ba would be only 6 kBq, activity comparable to that of ^40^K in an adult.

Moreover, the effective dose of [^18^F]F-PSMA-1007 applications lies between 4.4 and 5.5 mSv (Library-TENDL-2019. TENDL. 2019), the effective dose of 150 MBq [^68^Ga]Ga-PSMA-I&T is 3.0 mSv (Herrmann et al. 2015) and the effective dose of 200 MBq [^68^Ga]Ga-PSMA-11 is 4.7 mSv (Afshar-Oromieh et al. 2016), values that would be comparable to that of [^133^La]La-PSMA radioligands, even with the conservative assumption of no excretion.

Our dose estimation suffers from the assumption of a homogeneous activity distribution without excretion that would overestimate the expected dose. Furthermore, the dose contribution from ^133^Ba has a large variation due to the long half-life of ^133^Ba, but it is not expected that the maximum effective dose will occur due to the short life expectancy of the patients.

### Irradiation parameters optimization

A clear disadvantage of using a low [^134^Ba]BaCO_3_ amount is the large uncertainty in the saturation yield observed, which can be explained by the loading and unloading of the target material into the disc. In particular, the difference in loading ± 1 mg represents a ± 43 MBq/µA difference in the ^133^La theoretical saturation yield. This key uncertainty source suggests that the saturation yields obtained for both energy windows studied lie within their uncertainty range, although a slightly higher yield can be expected for the proton incident energy of 21 MeV. However, the lower energy window was selected as the most attractive to perform the irradiations due to the following reasons. Firstly, lower proton energy means less activation products in the backing disc, in addition to lower heat generation which is favorable to better cool the target. Secondly, in case the used 600 µm aluminum degrader suffers an unexpected reduction, the lower energy extraction energy from the cyclotron would still assure no ^132^La co-production.

On the other hand, the main advantage of such a target is that higher proton currents can be endured. In fact, previous studies using either 200 mg pure [^135^Ba]BaCO_3_ or 200 mg [^135^Ba]BaCO_3_ with aluminum in a ratio 1:2, were irradiated only with proton currents of up to 10 µA and 20 µA, respectively (Nelson et al. 2021; Pedersen et al. 2023). In this case, the use of higher proton currents compensates the lower yield of thinner targets. In addition, lower target masses also lead to higher separation factors and a cleaner product, that results of primordial importance considering that the macropa chelator is also able to form complexes with the divalent barium cation (Reissig et al. 2020) and thus reduce the AMA of the product [^133^La]LaCl_3_.

Moreover, the stability of the target configuration was proved by the 2 h irradiation. Based on the performed thermal simulations, the temperature in the [^134^Ba]BaCO_3_ is quite below this material melting point, which lies at 811 °C. As well, in contrast to calcium carbonate, barium carbonate can be melted without decomposition, since the latter only begins at 986 °C (Arvanitidis et al. 1996). In fact, the aluminum foil would melt before the barium carbonate melting or decomposition. In addition, the target is not sealed, which allows potential CO_2_ generation to exit the target.

Of note, the 2-h irradiation could be further extended to 4 h (about one ^133^La half-life), leading to activities of up to 18 GBq at EOB, which followed by an efficient radiochemical purification would account for 15 GBq at EOP, considering the involved times and the separation efficiency.

### Radiochemical separation

Although all the separation columns led to a successful ^133^La purification, the two resins, bDGA and TK222, affording the sharpest elution curves, and therefore reduced elution volumes, were selected. Our method proved to be straight-forward, obtaining a ready-to-label [^133^La]LaCl_3_ product after only 20 min. In addition, one further advantage of using the TK222 resin for the purification is the reduction in the acid concentrations used, increasing the pH of the obtained product which is favorable for the radiolabeling and reduces the cost of the needed acid for the washing fraction. This latter point is particularly relevant considering the large volume used for this.

### Product characterization

No major differences were observed in the RNP of the product fractions obtained from the different resins and no conclusion could be drawn. This may be attributed to the fact that the main radionuclide impurity is a radiolanthanum isotope, ^135^La, of which the content is independent of the radiochemical purification.

Differences in radiolabeling with the ^133^La purified using the different resins can be explained by the different content of cold metals in solution. The content of barium and lead results particularly important. In fact, the product obtained by purification with the TK222 resin proved a lower metallic content and it was reflected in a higher AMA.

On the other hand, the relatively high Pb^2+^ concentration in the product [^133^La]LaCl_3_ can be explained by the manual processing method, since it was performed with open vials surrounded of lead shielding and containers. The lead content resulted quite variable among the production batches and in most cases, it did not seem critical (< 2 µg Pb per GBq ^133^La). However, this problem could be addressed with a cassette-like production in a closed module, reducing the stable lead input.

To sum up, high ^133^La activities can be produced formulated in a ready-for-labeling [^133^La]LaCl_3_ solution. The method here presented proved both a high RNP and AMA, which at EOP are over 99.5% and 120 GBq/µmol, respectively. Extrapolating these values to 10 h after EOP, the RNP is still over 98% while the AMA drops to 20 GBq/µmol; quality comparable to that used for diagnostic radiopharmaceuticals in clinical studies, e.g. ^64^Cu- or ^68^Ga-labeled tracers (Pfeifer et al. 2012; Lin et al. 2020).

### Radiolabeling of PSMA ligands and stability study

Successful radiolabeling of PSMA-617 and mcp-M-PSMA with molar activities similar to those calculated as the AMA was carried out further evidenced the quality of the produced [^133^La]LaCl_3_. In particular, the highest affinity of lanthanum for the macropa chelator in comparison to DOTA was also observed, obtaining higher radiochemical conversions using the same radiolabeling conditions (molar activity, temperature and time).

A slightly higher serum stability of the [^133^La]La-macropa complex in comparison to that of the [^133^La]La-DOTA complex was quantified. In addition, the results obtained for [^133^La]La-PSMA-617 are consistent with previously published results for the [^135^La]La-DOTA complex (Pedersen et al. 2023). Moreover, it is particularly interesting to observe that the free [^133^Ba]Ba^2+^, daughter of ^133^La, could be differentiated from the free parent radionuclide, [^133^La]La^3+^. This enables detection of free ^133^Ba traces in the [^133^La]La-PSMA-617 probes in contrast to the ones corresponding to [^133^La]La-mcp-M-PSMA—this can be explained due to the higher potential of macropa to complex barium. While DOTA very poorly chelates this alkaline earth metal, it has been already shown that macropa is able to complex barium to some extent (Reissig et al. 2020). Then, the presence of the [^133^Ba]Ba-mcp-M-PSMA radioligand reduces the content of free ^133^Ba observed in the radio-TLC. Considering the initial 50 MBq of ^133^La, after 25 h about 600 kBq of ^133^La, 2 kBq of ^133^Ba (decay product) and about 1 kBq of ^133m^Ba (radionuclide impurity) are expected.

## Conclusions

In the present work we have demonstrated the possibility to up-scale the cyclotron-based ^133^La production, increasing the activity yields by ca. ten-fold and reaching 10.7 GBq at EOB. The used target design, based on simplicity and intending to reduce costs, showed a satisfactory performance for high proton current intensities. Additionally, four different chromatographic resins suitable for barium-lanthanum separation were tested and two optimized methods were obtained, which within 20 min led to 1 mL of a ready-for-labeling solution containing over 94% of the ^133^La activity. This could enable potential kit-like radiolabeling. The obtained [^133^La]LaCl_3_ presented high quality for radiolabeling, with RNP of over 99.5% and AMA of over 120 GBq/µmol at EOP. These obtained values are comparable to those often used in the clinics. Stability of ^133^La-PSMA-radioligands, namely [^133^La]La-PSMA-617 and [^133^La]La-mcp-M-PSMA, were tested and proved to be higher than 98% and 99%, respectively, after 24 h (over six ^133^La half-lives). Moreover, dosimetry estimations assessed the needed activity for a patient PET acquisition and led to activities between 350 and 750 MBq. This means that it is possible to produce the activity needed for over 15 patients with our current method, considering the shipping and radiolabeling times involved. In addition, first estimations have shown that the effective dose in patients for imaging with ^133^La would lie in the 2.1–4.4 mSv range, which is comparable to other positron-emitting radionuclides. We believe that further promoting ^133^La-macropa based radiopharmaceuticals for diagnosis will enable its safe translation into the clinics.

## Data Availability

The datasets used and/or analyzed during the current study are available from the corresponding author on reasonable request.

## Abbreviations

ACSI: Advanced Cyclotron System Inc
AMA: Apparent molar activity
DFO: Deferoxamine
DGA: Diglycolamide
DOTA: 2,2′,2″,2‴-(1,4,7,10-Tetraazacyclododecane-1,4,7,10-tetrayl)tetraacetic acid
DOTMP: 2,2′,2″,2‴-(1,4,7,10-Tetraazacyclododecane-1,4,7,10-tetrayl)tetraphosphonic acid
DOTPA: 2,2′,2″,2‴-(1,4,7,10-Tetraazacyclododecane-1,4,7,10-tetrayl)tetrapropionic acid
DTPA: Diethylenetriaminepentaacetatic acid
EC: Electron capture
EDTA: Ethylenediaminetetraacetic acid
EOB: End of bombardment
EOP: End of purification
HZDR: Helmholtz–Zentrum Dresden–Rossendorf
ICP-MS: Inductively coupled plasma mass spectrometry
iTLC: Instant thin layer chromatography
LET: Linear energy transfer
MAE: Meitner–Auger electron
mcp: Macropa
MRI: Magnetic resonance imaging
PET: Positron emission tomography
PSMA: Prostate specific membrane antigen
RCY: Radiochemical yield
RNP: Radionuclide purity
SG: Silica gel
TAT: Targeted alpha therapy
TETA: 1,4,8,11-Tetraazacyclotetradecane-1,4,8,11-tetraacetic acid
TETPA: 1,4,8,11-Tetraazacyclotetradecane-1,4,8,11-tetrapropionic acid
VKTA: Radiation protection, analytics and disposal
